# Disruption of *MAP7D1* Gene Function Increases the Risk of Doxorubicin-Induced Cardiomyopathy and Heart Failure

**DOI:** 10.1155/2021/8569921

**Published:** 2021-07-15

**Authors:** Li-Ping Li, Jing Zhong, Mei-Hang Li, Yuan-Chao Sun, Yu-Juan Niu, Chuan-Hong Wu, Jian-Feng Zhou, Nadine Norton, Zhi-Qiang Li, Yong-Yong Shi, Xiao-Lei Xu, Yong-He Ding

**Affiliations:** ^1^School of Basic Medicine, The Biomedical Sciences Institute of Qingdao University (Qingdao Branch of SJTU Bio-X Institutes), Qingdao University, Qingdao 266021, China; ^2^The Affiliated Hospital of Qingdao University & The Biomedical Sciences Institute of Qingdao University (Qingdao Branch of SJTU Bio-X Institutes), Qingdao University, Qingdao 266021, China; ^3^Key Laboratory of Marine Drugs (Ocean University of China), Chinese Ministry of Education, School of Medicine and Pharmacy, Ocean University of China, Laboratory for Marine Drugs and Bioproducts, Pilot National Laboratory for Marine Science and Technology (Qingdao), Qingdao 266003, China; ^4^Department of Cancer Biology, Mayo Clinic, Jacksonville, FL 32224, USA; ^5^Department of Biochemistry and Molecular Biology, Mayo Clinic, Rochester, MN 55902, USA

## Abstract

Doxorubicin is a cornerstone chemotherapeutic drug widely used to treat various cancers; its dose-dependent cardiomyopathy, however, is one of the leading causes of treatment-associated mortality in cancer survivors. Patients' threshold doses leading to doxorubicin-induced cardiomyopathy (DIC) and heart failure are highly variable, mostly due to genetic variations in individuals' genomes. However, genetic susceptibility to DIC remains largely unidentified. Here, we combined a genetic approach in the zebrafish (*Danio rerio*) animal model with a genome-wide association study (GWAS) in humans to identify genetic susceptibility to DIC and heart failure. We firstly reported the cardiac and skeletal muscle-specific expression and sarcomeric localization of the microtubule-associated protein 7 domain-containing protein 1b (Map7d1b) in zebrafish, followed by expression validation in mice. We then revealed that disruption of the *map7d1b* gene function exaggerated DIC effects in adult zebrafish. Mechanistically, the exacerbated DIC are likely conveyed by impaired autophagic degradation and elevated protein aggregation. Lastly, we identified 2 *MAP7D1* gene variants associated with cardiac functional decline and heart failure in cancer patients who received doxorubicin therapy. Together, this study identifies *MAP7D1* as a clinically relevant susceptibility gene to DIC and heart failure, providing useful information to stratify cancer patients with a high risk of incurring severe cardiomyopathy and heart failure after receiving chemotherapy.

## 1. Introduction

Anthracyclines are the milestone of chemotherapy drugs used to treat a variety of tumors. To date, anthracyclines remain among the most potent anticancer chemotherapy drugs ever, and up to 60% of cancer patients received anthracycline-based chemotherapy [1]. Doxorubicin is one of the most important types of anthracyclines and was listed as an essential cytotoxic medicine by the World Health Organization [2]. Its dose-dependent cardiac dysfunction and heart failure, however, drastically compromised its more widely clinical usage [3]. While accumulated dose represents the major risk factor for the severity of doxorubicin-induced cardiomyopathy (DIC), genetic makeup is also well recognized to significantly contribute to the highly variable threshold doses leading to DIC among individual patients [1].

To identify genetic susceptibility to DIC and heart failure, strategies such as the candidate-gene approach and genome-wide association studies (GWASs) have been mostly used [1]. While some candidate-gene approaches focused on genes involving pharmacokinetics and/or metabolisms of doxorubicin that mostly affect the acute cardiomyopathy which are usually clinically manageable, multiple unbiased GWASs have been used to identify genes/variants associated with the chronic and irreversible side effects of DIC [4, 5]. These chronic DIC-associated genes usually exhibit abundant expression in the heart organ and play primary roles in cardiac remodelling. Consistently, genetic variants in cardiomyopathy-causative genes, such as truncation variants in the titin gene, significantly increased the risk of anthracycline drug-associated cardiomyopathy and adverse cardiac events [5, 6].

Due to its amenability to high-throughput genetic and compound screening, zebrafish (*Danio rerio*) has emerged as a highly prolific vertebrate animal model to study human cardiac diseases [7–9]. Surprisingly, many cardiac electrophysiological behaviors of zebrafish have higher conservation than mouse with humans [7]. To study the genetic basis of DIC, we previously established a method to model DIC in adult zebrafish through injection of a single bolus of doxorubicin (20 *μ*g/g) intraperitoneally [10]. Four weeks after doxorubicin injection, adult zebrafish manifested key features of chronic cardiomyopathy and heart failure similar to human patients. We recently also developed a gene-breaking transposon- (GBT-) based gene-trap system in zebrafish that enables to disrupt gene function highly efficiently (>99% at the RNA level) while simultaneously reporting the endogenous gene expression and subcellular localization of tagged proteins [11, 12]. Based on this unique gene expression and subcellular localization reporting system, we collected 44 GBT cardiac mutants in which each mutant harbors a cardiac expressed gene disruption [13].

Here, we focused on the cardiac and skeletal muscle-specific expressing *GBT239* mutant that tagged to the microtubule-associated protein 7 domain-containing protein 1b (*map7d1*b) and reported its previously unrecognized function in doxorubicin-induced cardiac damage and heart failure. To further provide clinical relevance, we analyzed a reported human GWAS dataset conducted in cancer patients who received doxorubicin therapy and identified two *MAP7D1* gene variants associated with cardiac function decline and heart failure in humans.

## 2. Materials and Methods

### 2.1. Animals and Ethic Statements

The zebrafish (*Danio rerio*) were maintained under a 14-hour light/10-hour dark cycle at 28.5°C. The gene-transposon breaking (GBT) *239/map7d1b* mutant was generated previously (http://zfin.org/) [12]. All animal procedures were conducted in accordance with the Institutional Guidelines for Animal Use and Care of Qingdao University, following the *Guide for the Care and Use of Laboratory Animals* published by the US National Institutes of Health (NIH Publication No. 85-23, revised 1996).

### 2.2. Genotyping PCR

The *GBT239/map7d1b* mutant fish were identified by genotyping PCR, using genomic DNA isolated from the tail fin as a template as reported previously [14]. The *map7d1b* gene-specific primer pairs (primer-F1) 5′-CAACTGGAGAGACAGAGACGTG-3′ and (primer-R1) 5′-ATCGAGGGTTTGGAGGATCGTG-3′ combined with the GBT transposon sequence-specific primer (primer-RP2) 5′-GTACAGTAATCAAGTAAAATTACTCA-3′ were used.

### 2.3. Determining Knockdown Efficiency

Quantitative real-time RT-PCR was used to determine the *map7d1b* gene knockdown efficiency in the *GBT239/map7d1b* mutant accordingly [14]. Briefly, total RNA was isolated using the TRIzol reagent (Invitrogen), and ~1 *μ*g of total RNA was used for cDNA synthesis with the SuperScript III cDNA kit (Invitrogen). The RT-PCR primers 5′-AAAACTGACGCTGTCAGAC-3′ and 5′-TCTGTGATGAGGGAGGAGTC-3′ were designed in the two neighboring exons flanking the transposon insertion site. When the two exons were properly spliced together, a native “wild-type” product was detected, while in the mutant, the proper splicing event was interrupted by the insertion vector, yielding an aberrant splicing product.

### 2.4. Doxorubicin Injection

The *GBT239* homozygous fish and WT controls at 3 months were fasted for 24 h before subjected to doxorubicin injection. The amount of injected doxorubicin (Sigma) was determined based on body weight. After fish were euthanized by incubation with 0.016% tricaine for 3 min, a single dose at 20 *μ*g/g body weight of doxorubicin dissolved in 5 *μ*L Hank's buffer was injected intraperitoneally using a NanoFil 10 *μ*L syringe (World Precision Instruments Inc). Cardiac function indices and swimming capacity were measured at 4 weeks postdoxorubicin injection. Fish survival was monitored daily for 2–4 months after doxorubicin injection.

### 2.5. Echocardiography

Echocardiography measurement was carried out using the Vevo 3100 high-frequency imaging system equipped with a 50 MHz (MX700) linear array transducer (FUJIFILM VisualSonics Inc.) accordingly [15]. Briefly, adult zebrafish at appropriate stages were anesthetized in tricaine (0.016%) for 5 minutes, placed ventral side up into a sponge. The MX700 transducer was placed above the zebrafish to provide a sagittal imaging plane for the heart. Image quantification was performed using the VevoLAB workstation. For each index on individual fish, measurements were performed on 3 independent cardiac cycles to acquire average values.

### 2.6. Western Blot Analysis

Heart tissues from BL6 WT mice or doxorubicin-treated fish were homogenized in RIPA buffer using a Bullet Blender (Next Advance). Lysate samples were denatured at 95°C for 10 min and subjected to Western blotting. Anti-Map7d1 (1 : 1000, ThermoFisher Scientific, catalog **#** BS-9314R), anti-LC3 (1 : 3000, Novus Biologicals, catalog # NBP100-2331), anti-Grp78 (1 : 2000, Novus Biologicals LLC, catalog # NBP-06274), anti-ubiquitin (1 : 1000, ThermoFisher Scientific, catalog # PA5-17067), and anti-Gapdh (1 : 5000 Santa Cruz Biotechnology, catalog # sc-25778) antibodies were used.

### 2.7. Hematoxylin and Eosin (H&E) Staining

Heart tissues were dissected from individual adult fish at the designed stages after euthanasia by incubation with 0.032% tricaine for 10 minutes. Isolated hearts were immediately fixed in 4% PBS-buffered formaldehyde and paraffin-embedded and sectioned. The sectioned heart tissues were then stained in the hematoxylin for 3 min, followed by staining in the eosin staining for 30 s, with running tap water rinse between each for 5 min. The density of the trabecular muscle was quantified using ImageJ software.

### 2.8. Terminal Deoxynucleotidyl Transferase- (TdT-) Mediated dUTP Nick End Labeling (TUNEL)

Hearts were harvested from fish after being euthanized by incubation with 0.032% tricaine for 10 minutes. Cryostat-sectioned fish ventricles (8 *μ*m) were stained with the In Situ Cell Death Detection Kit, Fluorescein (Roche Applied Science), according to the manufacturer's protocol.

### 2.9. Swimming Tunnel Assay

The swimming tunnel protocol was conducted using a swim tunnel respirometer (Mini Swim 170, Loligo Systems, Denmark) according to the previous reports [15]. Both *GBT239* mutant and WT fish after doxorubicin injection were fasted for 24 h prior to swimming capacity measurement. Fish were loaded into the swimming tunnel and acclimated at a lower speed of 9 cm/s (200 rpm) of flowing water for 20 min. Water speed was then increased in steps of 8.66 cm/s (100 rpm) (*U*ii) at 150 s intervals (*T*ii) until all fish were exhausted and failed to resume swimming from a downstream screen. The highest water speed (*U*i) against which fish were able to complete the 150 s swim test and the swimming duration time (*T*i) in the next 150 s period were recorded for each fish. *U*crit was defined as the maximum water speed that fish were able to swim against while maintaining their position, which was calculated with the following formula: *U*crit = *U*i +*U*ii × (*T*i/*T*ii) and normalized to fish body length (BL) for intergroup comparisons.

### 2.10. To Identify MAP7D1 Variants Associated with Cardiac Dysfunction and Heart Failure

The previously reported GWAS dataset conducted in 1191 breast cancer patients who received adjuvant doxorubicin therapy from the N9831 phase III clinical trial was mined for the *MAP7D1* variants associated with cardiac function decline and heart failure [16]. A linear regression analysis of the maximum decline in left ventricular ejection fraction (LVEF) was used, adjusting for age, baseline LVEF, and antihypertensive medications. Under an additive model, the effect size (beta) of variants associated with the maximum decline in LVEF was calculated.

### 2.11. Statistical Analysis

Unpaired 2-tailed Student *t*-test was used to compare between mutants and WT controls. For survival analysis, log rank tests were used. *P* values less than 0.05 were considered statistically significant. Results are presented as the mean ± standard deviation (SD). All statistical analyses were conducted with the GraphPad Prism 7 software.

## 3. Results and Discussion

### 3.1. A Transposon Insertion Disrupted the *map7d1b* Gene That Is Predominantly Expressed in the Cardiac and Skeletal Muscle

**Figure 1:**
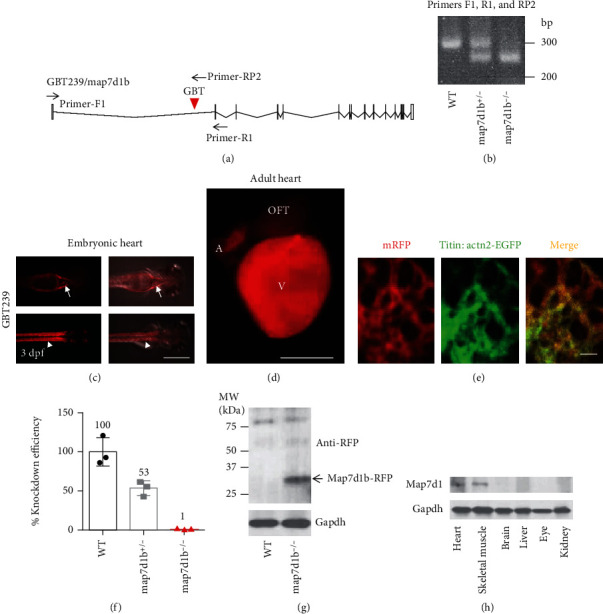
A transposon insertion in the *GBT239* mutant disrupted the *map7d1b* gene that is predominantly expressed in the cardiac and skeletal muscle. (a) Insertional position of a gene-break transposon (GBT) element into the first intron of the *map7d1b* gene in the *GBT239* mutant. (b) Representative DNA gel images of PCR genotyping for identifying *GBT239* heterozygous (*map7d1b^+/-^*) and *GBT239* homozygous (*map7d1b^−/−^*) mutant alleles. (c) Imaging the *GBT239* mutant at 3 days postfertilization (dpf) reported the cardiac (arrows) and skeletal muscle (arrowheads) specific expression of the tagged Map7d1b protein. Scale bar: 0.5 mm. (d, e) Imaging the *GBT239* adult heart indicated ventricle enriched expression of the tagged Map7d1b protein (d), which largely overlapped with the EGFP signal in the sarcomere reporter line *Tg(titin:actn2-EGFP)* (e). V: ventricle; A: atrium; OFT: outflow tract. Scale bar in (d): 1 mm. Scale bar in (e): 20 *μ*m. (f) Quantitative RT-PCR analysis of the native *map7d1b* transcript disruption in the *GBT239/map7d1b* mutant. (g) Western blotting analysis of the Map7d1b-RFP fusion protein in the *GBT239/map7d1b* mutant. Arrow indicates the predicted size of Map7d1b-RFP fusion protein. (h) Western blotting analysis of the Map71b protein in different mouse tissues.

We hypothesized that susceptibility genes associated with chronic doxorubicin-induced cardiomyopathy (DIC) would exhibit abundant expression in the heart organ. Taking advantage of the unique fluorescence reporting system integrated into the gene-breaking transposon (GBT) system in zebrafish, we recently reported a collection of zebrafish insertional cardiac mutants in which each mutant harbors a cardiac expressed gene disruption [11–14]. Here, we reported the *GBT239* mutant which is linked to the microtubule-associated protein 7 domain-containing protein 1b (*map7d1*b) gene (Figures 1(a) and 1(b)). The *GBT239/map7d1b* mutant exhibited cardiac and skeletal muscle-specific expression patterns (Figure 1(c)). More detailed fluorescent image analysis revealed that the *GBT239* mutant tagged Map7d1b protein was mostly localized to the sarcomere in the adult zebrafish heart, based on its overlapping patterns with the EGFP signal in the sarcomere reporter line *Tg(titin:actn2-EGFP)* (Figures 1(d) and 1(e)) [17]. At the transcript level, about 47% of the native *map7d1b* transcript was disrupted in the *GBT239* heterozygous (*map7d1b^GBT239/+^*, also termed *map7d1b^+/-^*) and 99% was disrupted in the *GBT239* homozygous (*map7d1b^GBT239/GBT239^*, also termed *map7d1b^−/−^*) mutant (Figure 1(f)). Disruption of Map7d1b protein in the *map7d1b^−/−^* mutant was further determined by detection of the Map7d1b-RFP fusion protein (Figure 1(g)). The specific cardiac and skeletal muscle expression patterns of Map7d1 were conserved in the mouse (Figure 1(h)).

### 3.2. Disruption of the *map7d1b* Gene Exacerbated Doxorubicin-Induced Cardiac Dysfunction and Heart Failure

**Figure 2:**
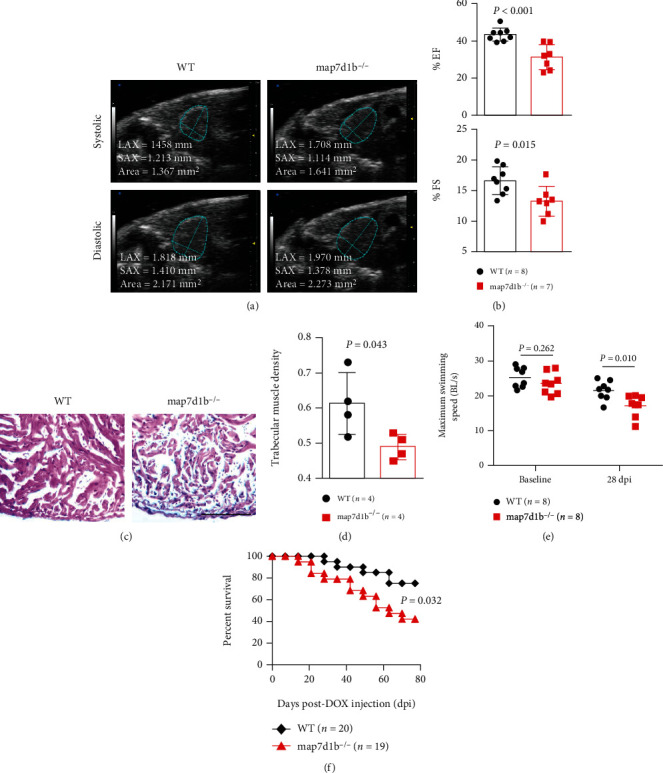
Disruption of *map7d1b* gene in the *GBT239* homozygous mutant exacerbated doxorubicin-induced cardiac dysfunction and heart failure. (a) Shown are examples of echocardiography images extracted from movies of beating hearts in WT controls and *GBT239/map7d1b* homozygous (*map7d1b^−/−^*) mutants at systole (upper panel) and diastole (lower panel) contraction. (b) Quantification of cardiac function indices of ejection fraction (EF) and fractional shortening (FS) measured by echocardiography in the *map7d1b^−/−^* mutant compared to WT control at 4 weeks postdoxorubicin injection. *n* = 7-8, Student's *t*-test. (c) Representative images of H&E staining of the ventricles at 4 weeks postdoxorubicin injection. Scale bar: 100 *μ*m. (d) Quantification of trabecular muscle density in the *map7d1b^−/−^* mutant compared to WT controls at 4 weeks postdoxorubicin injection. *n* = 4, Student's *t*-test. (e) Maximum swimming speed of the *map7d1b^−/−^* mutant compared with the WT control at both baseline and 28 days postdoxorubicin injection (dpi). *n* = 8, Student's *t*-test. (f) Kaplan-Meier survival curves of WT and *map7d1b^−/−^* mutant zebrafish injected with a single bolus of 20 *μ*g/gram body mass (gbm). The *map7d1b^−/−^* mutant had a significantly reduced survival than WT controls. *n* = 19–20, log rank test.

Despite 99% knockdown of the *map7d1b* gene transcript, the *GBT239* homozygous mutant fish (*map7d1b^−/−^*) appeared indistinguishable from their wild-type (WT) siblings under normal housing conditions (data not shown). However, upon the injection of a single bolus of 20 *μ*g/gram body mass (gbm) doxorubicin intraperitoneally, the *map7d1b^−/−^* mutant fish exhibited significantly declined ejection fraction (EF) and fractional shortening (FS) compared to the WT controls, as measured by a noninvasive high-frequency echocardiography imaging system (Figures 2(a) and 2(b)). Histology analysis revealed obvious myofibril loss and significant damage of the trabecular muscle in the doxorubicin-injected *map7d1b^−/−^* mutant fish hearts (Figures 2(c) and 2(d)). As an important clinical index for heart failure in human patients, the swimming capacity of *map7d1b^−/−^* mutant fish was also significantly reduced (Figure 2(e)). Furthermore, the *map7d1b^−/−^* mutant fish exhibited significantly increased mortality, and about 60% of the *map7d1b^−/−^* mutant fish died within 80 days postdoxorubicin injection (Figure 2(f)). Taken together, these results demonstrated that disruption of *map7d1b* gene function exacerbated doxorubicin-induced cardiac dysfunction and heart failure in the adult zebrafish animal model.

### 3.3. Disruption of *map7d1b* Gene Led to Increased Apoptotic Cardiomyocyte Death Concurrent with Impaired Autophagy and Elevated Protein Aggregation upon Doxorubicin Stress

**Figure 3:**
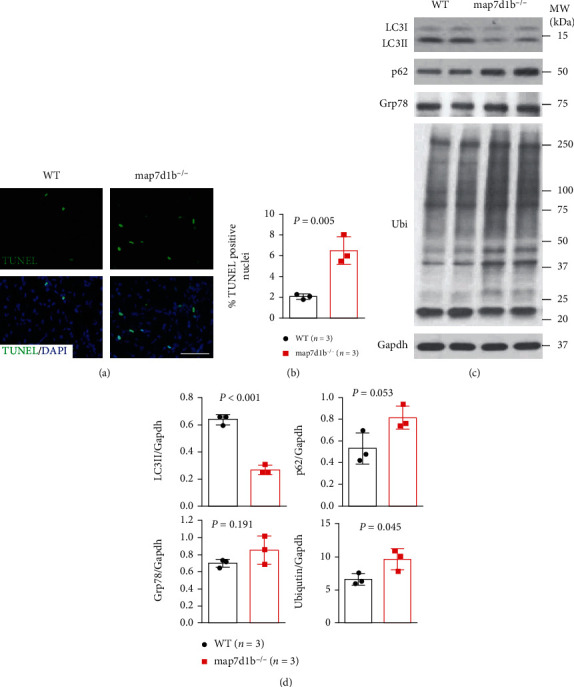
The *GBT239/map7d1b* homozygous mutant exhibited increased apoptotic cardiomyocyte death concurrent with impaired autophagy and elevated protein aggregation upon doxorubicin stress. (a, b) Representative images of the TUNEL assay (a) and quantification of the percentage of TUNEL-positive nuclei in GBT239/map7d1b homozygous mutant (map7d1b^−/−^) compared to WT controls at 4 weeks postdoxorubicin injection. *n* = 4, Student's *t*-test. Scale bar: 20 *μ*m. (c, d) Representative Western blot images (c) and quantification analysis (d) of the expression levels of autophagy molecular markers LC3II and p62 (SQSTM1), ER stress marker glucose-regulated protein 78 (Grp78), and ubiquitin aggregated protein examined in the heart tissues isolated from the map7d1b^−/−^ mutant compared to WT control at 4 weeks postdoxorubicin injection. *n* = 3, Student's *t*-test.

We then sought to further investigate underlying pathophysiologic mechanisms on how disruption of *map7d1b* gene function incurred exaggerated DIC effects. At the cellular level, we carried out the terminal deoxynucleotidyl transferase-mediated dUTP nick end labeling (TUNEL) assay to evaluate the cardiomyocyte apoptosis, which is considered a major index for DIC in human patients. A significantly increased TUNEL index was detected in the *map7d1b^−/−^* mutant fish hearts compared to WT controls (Figures 3(a) and 3(b)). As impaired autophagy flux was recently proposed to contribute to the DIC in the mouse model [18], we assessed the autophagy activities in *map7d1b^−/−^* mutant hearts. We found that the protein level of LC3II, a molecular marker for autophagy formation, was significantly reduced in the *map7d1b^−/−^* mutant hearts compared to WT controls (Figures 3(c) and 3(d)). On the other hand, the level of p62 protein, which represents the autophagy degradation activity, was significantly increased in the *map7d1b^−/−^* mutant hearts. Furthermore, increased protein aggregation, as revealed by elevated protein ubiquitination, was detected in the *map7d1b^−/−^* mutant hearts. In contrast, the endoplasmic reticulum (ER) stress molecular marker glucose-regulated protein 78 (Grp78) was not statistically changed. Taken together, these data suggested that increased apoptotic cardiomyocyte death concurrent with impaired autophagy and elevated protein aggregation most likely convey the exaggerated DIC effects due to *map7d1b* gene disruption.

### 3.4. GWAS Evidence to Support the *MAP7D1* as a Genetic Susceptibility Gene to DIC and Heart Failure

To seek clinical relevance of the human *MAP7D1* gene susceptibility to DIC, we mined a dataset from a reported genome-wide association study (GWAS) dataset conducted in 1191 breast cancer patients who received adjuvant doxorubicin therapy from the N9831 phase III clinical trial [16]. After using a linear regression analysis of the maximum decline in left ventricular ejection fraction (LVEF), adjusted for age, baseline LVEF, and antihypertensive medications, we identified 2 rare variants in intron of the *MAP7D1* locus, rs272825 and rs272832, which are associated with a decline in LVEF with the low *P* values of 5.63 × 10^−5^ and 5.70 × 10^−5^, respectively (Table 1). Under an additive model, the effect size (beta) of these 2 variants suggests that patients with one copy of the risk allele would have on average a maximum decline in LVEF of -27.24 points (Table 1). As for patients' LVEF monitored throughout the clinical trial with standard interventions for the decline in heart function, 17 of them experienced irreversible congestive heart failure (CHF), while the number of patients with CHF was too small for GWAS analysis, within this subgroup, the associated allele in the primary model was significantly enriched for the *MAP7D1* rare variants (2.9% vs. 0%) with the *P* value of 0.014 (Fisher exact test). Thus, the *MAP7D1* gene was also significantly associated with patients with the worst cardiac outcome. Together with its association with decline in LVEF and CHF and large effect size in the quantitative analysis, these GWAS data, despite the limitation of lacking an anthracycline alone treatment arm, supported *MAP7D1* as a clinically significant susceptibility gene to DIC and heart failure.

## 4. Discussion

### 4.1. To Combine the Power of Zebrafish Genetics and a Human GWAS for Identifying Susceptibility Gene to Doxorubicin-Induced Cardiomyopathy

In the era of individualized medicine, genome sequencing for every patient may become routinely available and GWASs are expected to generate vast genomic variants/single nucleotide polymorphisms (SNPs) associated with specific disease phenotypes such as susceptibility to doxorubicin-induced cardiomyopathy (DIC) [1]. However, the major challenges in using the GWAS technique alone are the difficulty in defining the genotype-phenotype relationships and lacking functional validation for the vast candidate genetic loci identified [19]. Because of its amenability to high-throughput genetic and compound screening, zebrafish has emerged as an attractive animal model for systematic gene discovery in cardiovascular diseases [20]. Here, based on a unique endogenous gene expression-reporting system developed in the zebrafish animal model, we reported the cardiac enriched expression of *map7d1b* gene, followed by functional characterization that disruption of *map7d1b* gene function predisposed and exaggerated the DIC effects. Through analyzing a reported GWAS dataset [16], we identified 2 variants in the human *MAP7D1* gene significantly associated with decline of cardiac function and heart failure in cancer patients who received the doxorubicin chemotherapy. These results provide compelling evidence to support that mutations in *MAP7D1*gene would increase the risk of DIC and heart failure in humans. Thus, a major novelty of this study is to combine the power of zebrafish genetics and a human GWAS for both identification and functional elucidation of *MAP7D1* as a clinically relevant susceptibility gene to DIC and heart failure. The feasibility of this approach was further supported by recent studies that reported *RXRA* as a genetic factor associated with anthracycline-induced cardiotoxicity (AIC) [13, 15]. This approach, we believe, can be readily applied to study other AIC genes as well.

### 4.2. Functional Mechanisms Underlying the *map7d1b* Gene in Doxorubicin-Induced Cardiomyopathy

**Figure 4:**
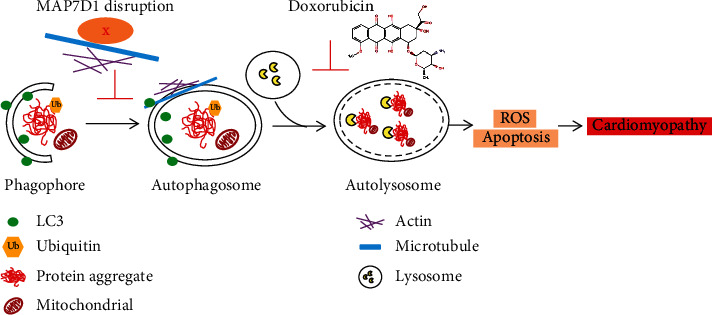
Working model of MAP7D1 in doxorubicin-induced cardiomyopathy. Doxorubicin causes cardiotoxicity by inhibiting autolysosome formation, resulting in elevated ROS and cardiomyocyte apoptosis. Disruption of MAP7D1 protein function further impaired autophagosome formation and led to accumulation of toxic protein aggregation, thus exacerbating doxorubicin-induced cardiomyopathy and heart failure.

The pathophysiological mechanisms by which anthracycline caused cardiomyocyte cell death and cardiomyopathy were to produce overwhelmed oxidative stress that damaged various cellular components [21]. Autophagy *is a* self-rejuvenation cellular process by clearing damaged organelles and recycling cellular components to maintain cellular and tissue homeostasis, which has emerged as one of the major signaling pathways linked to DIC [18, 22]. MAP7D1 belongs to the microtubule-associated protein (MAP) family known to bind microtubules and play essential roles in coordinating many crucial steps of autophagosome formation [23]. As assembly of autophagy machinery relies on proper sequestration, recruitment, and interaction of many factors with the microtubules, it is plausible to speculate that disruption of factors associated with the microtubule dynamics, such as the Map7d1b, would impair the orchestrate movement of preautophagosomal structures and subsequent maturity of autophagosome and lysosome. Because of the vital role of autophagic machinery in maintaining protein homeostasis, autophagy impairment could impose extra stress in the protein quality control system and lead to accumulation of unfolded protein aggregation. Consistently, elevated ubiquitinated protein aggregation was detected in the *map7d1b^−/−^* mutant hearts. Thus, this study suggests function of Map7d1b as a key microtubule-associated factor involved in autophagy assembly and protein turnover in doxorubicin-induced cardiac damage and heart failure (Figure 4).

## 5. Conclusions

This study combined the power of forward genetic study in the zebrafish animal model and a GWAS in humans to identify *MAP7D1* as a clinical relevant susceptibility gene to DIC. Disruption of *MAP7D1* gene function would increase the risk of cancer patients to developing cardiomyopathy and heart failure after receiving doxorubicin chemotherapy. Mechanistically, the exacerbated DIC effects resultant from *MAP7D1* gene disruption were likely conveyed by impaired autophagy and elevated protein aggregation. Future research in the direction of identifying a more comprehensive gene/variant list underlying the genetic contributions to DIC can provide useful information toward developing better prediction tools to stratify cancer patients at high risk of incurring severe side effects of cardiomyopathy and heart failure after receiving anthracycline drug-based chemotherapy.

## Figures and Tables

**Table 1:** *MAP7D1* single nucleotide polymorphisms in patients with doxorubicin-induced cardiomyopathy.

			Association with maximum decline in LVEF					
SNP ID	Gene	Location	Beta	SE	L95	U95	*P* values	
rs272825	*MAP7D1*	Intron	-27.24	6.62	-40.21	-14.27	4.10*E* − 05	
rs272832	*MAP7D1*	Intron	-26.35	6.61	-40.20	-14.28	4.07*E* − 05	
			1000 genome annotation					
SNP ID	Gene	Location	Reference allele	Alternative allele	Asian AF	American AF	African AF	European AF
rs272825	*MAP7D1*	Intron	G	C	—	0.03	0.25	—
rs272832	*MAP7D1*	Intron	T	C	—	0.03	0.26	—

SNP: single nucleotide polymorphism; LVEF: left ventricular ejection fraction; SE: standard error; L95: lower bound 95; U95: upper bound 95; AF: allele frequency.

## Data Availability

All data used to support the finding of this study are available from the corresponding author upon request.
